# Potential Risk Factors for Varicose Veins with Superficial Venous Reflux

**DOI:** 10.1155/2014/531689

**Published:** 2014-09-11

**Authors:** Nazmiye Selçuk Kapısız, Tülin Uzun Kulaoğlu, Turgay Fen, Hasan Fahri Kapısız

**Affiliations:** ^1^Cardiovascular Surgery Department, Atatürk Education and Research Hospital, Bilkent, 06800 Ankara, Turkey; ^2^Radiology Department, Ankara Education and Research Hospital, 06340 Ankara, Turkey; ^3^Hematology Department, Ankara Education and Research Hospital, 06340 Ankara, Turkey; ^4^Cardiovascular Surgery Department, Ankara Yenimahalle State Hospital, 06170 Ankara, Turkey

## Abstract

The objective of the study is to evaluate a range of potential risk factors in the etiology of varicose veins with superficial venous reflux. Forty-nine patients attending a cardiovascular surgery clinic for the management of varicose disease between 2009 and 2010 were enrolled for the study. The age range of the patient group was 44,04 ± 15,05 years and female/male (F/M) ratio was 30/19. Twenty-six normal, healthy volunteers with the age of 40,94 ± 13,60 years and with the female/male ratio of 15/11 acted as control subjects. We investigated several parameters including body mass index, age, birth number > 1, standing for a long time (standing for 8 or more hours without taking a break), systemic diseases, family history, venous Doppler fındings, the levels of homocysteine, ferritin, vitamin B12, and hemoglobin, sedimentation rate, mean corpuscular volume, low density lipoprotein, and rheumatoid factor of the patient group and the control group. We also determined the contribution of the methylene tetrahydrofolate reductase 677 C>T and 1298 A>C gene polymorphisms and FV Leiden in both groups. In this small study, there appears to be no association between varicose veins and body mass index, smoking, type 2 DM, hypertension, family history, and birth number. A history of increased standing duration period (>8 hours) and rheumatoid factor positivity have association with varicose veins with superficial venous reflux.

## 1. Introduction

Varicose veins occur commonly in the general population but despite much research the etiology of venous disease is still poorly understood. Obesity, age, parity, standing for long times, and family history are risk factors. Incidence of varicose veins in adult population has been shown to vary among populations (between 10% and 60%) and to increase by age in various studies [1–6]. The main factors in the etiology of varicose vein are venous dilation and valvular insufficiency that are started by unknown factors [1–3, 7, 8].

A number of epidemiological studies have shown that, in addition to environmental factors, genetic mechanisms may play a role in determining susceptibility to vascular disease. In particular, abnormalities in the enzymes that control homocysteine metabolism have been shown to cause atherosclerotic vascular disease [9].

Since genetic mechanisms may play a role in determining susceptibility to vascular disease, we studied several mutations in patients with varicose veins to look for any association between varicose veins and homocysteine level, protein C, protein S, FV, FVIII, D-dimer, vitamin B12 level, folic acid level, MTHFR, FVR2, B fibrinogen, FV Leiden, and prothrombin mutations besides other possible factors.

The present study is planned to determine various risk factors and to analyze the methylenetetrahydrofolate reductase polymorphisms (*MTHFR-677 *and* MTHFR-1298*) and FV Leiden mutations in patients with primary varicose veins.

## 2. Materials and Methods

Forty-nine patients were enrolled for the study. The patients considered for the study were those attending Ankara Education and Research Hospital for the management of venous disease. All patients answered a standardized questionnaire. The following data were collected: age, height, weight, smoker or nonsmoker, personal and family medical history with a special focus on varicose veins and birth number >1 (women only), standing for a long time before, and additional diseases. Informed written consent was obtained from all patients and control volunteers fulfilling the entry criteria. This study protocol was approved by the Ethics Committee of Ankara Education and Research Hospital.

The diagnosis of primary varicose vein was performed by the combination of clinical and duplex scanning examination. All patients in the study had normal arterial examination. Every patient in the control and patient group had duplex examination. The exclusion criteria for the study were deep vein thrombosis, postthrombophlebitic syndrome, and recent infection.

There were 45 patients with superficial venous reflux, 2 patients with superficial and perforator venous reflux, and 2 patients with superficial venous reflux and healed venous ulcer. The reflux level was between grades 2 and 4 in the patient group. There were no patients with deep venous insufficiency in the study group. The effects of the grade and type of the reflux on the results were not studied in the paper. We could not evaluate the location of valvular reflux at the various levels, SFJ, upper thigh, midthigh, lower thigh, or below the knee, because of outpatient density in the Radiology Department. Since there were not enough patients in the study group, the differences in the gene polymorphism or the procoagulant parameters between C2 and C3 patients versus the control group were not analysed.

Twenty-six normal, healthy volunteers acted as control subjects. They had no symptoms or signs of arterial or venous disease in either limb. Venous Doppler findings of control subjects were normal. They did not have any superficial or deep venous reflux. The control subjects did not have any disease established by careful history, examination, and routine laboratory test including the levels of homocysteine, ferritin, vitamin B12, hemoglobin, sedimentation rate, mean corpuscular volume, low density lipoprotein, and rheumatoid factor. The patients in the observation group and the control group were similar regarding gender, body mass index, smoking habits, medical history, family medical history, and age. We obtained information about standing duration while obtaining history. We asked patients one by one about how long they stand up all through the day and recorded the duration of time in hours for each patient separately.

In the physical examination, subjects stood on a raised platform with their feet in three standard positions during inspection of the legs: facing towards the examiner with heels together and forefeet spread wide apart, facing away from the examiner in a similar position, and facing away from the examiner with feet parallel. They were asked to remain in a standing position for a minimum of two minutes before examination of their veins, to allow the blood to pool in the legs. Any scars or notable findings on the legs were recorded.

Real-time images of the common femoral, deep femoral, femoral, and popliteal veins were obtained before and after compression. Color and pulsed-wave Doppler sonography also was performed. All patients were evaluated with color duplex scanning in a warm, comfortable examination room by a radiology physician. The examination was performed using a GE Logic venous ultrasonography device. A 5–12 MHz linear transducer was used to measure the diameters of the great saphenous vein (GSV) and the small saphenous vein (SSV) and to rule out acute or chronic deep venous thrombosis (DVT) in supine position. Using color flow imaging in the longitudinal view, the valvular function of the GSV was evaluated at the SFJ, upper thigh, midthigh, lower thigh, and below the knee. The valvular function of the SSV was evaluated at the level of the popliteal fossa. Flow direction was noted with distal compression and release. The reflux was quantified based on valve closure time, with the Doppler spectral tracings obtained in a longitudinal plane. Reflux was defined as being present if the valve closure time was greater than 0.5 seconds. Examination for reflux was made with the patients standing, with upper body elevation of more than 45°, or in reverse Trendelenburg position.

### 2.1. Methods

The study was conducted in room temperature. Blood samples were taken from the antecubital vein by atraumatic puncture. Blood samples were collected into citrate tubes (for protein C, protein S, fibrinogen, and homocysteine) and centrifuged at 4000 rpm (revolutions per minute) for 4 min to obtain plasma and serum fractions. Samples were then stored at 22°C until being assayed. Before assay, the samples were thawed at room temperature.

### 2.2. Proteins C and S

The assays were performed by international laboratory supported by Beckman Coulter. Quantitative determination of functional protein C depended on the prolongation of activated partial thromboplastin time. Protein S was determined in accordance with the inhibition of activated factor V. The normal ranges of protein C and S antigens in plasma were 70–130% and 65–140%, respectively.

### 2.3. Homocysteine

Serum total homocysteine levels were determined by chemiluminescence immunoassay.

### 2.4. Factor XIII, Factor XV, and Factor VIII

FXIII testing was performed using commercially available kits according to the manufacturer's instructions. The assay kit is designed to quantitate the true functional FXIII and FXIIIa activity in plasma by measuring transglutaminase activity. In this assay, the thrombin-activated FXIIIa from the patient plasma binds to a substrate coated plate. Next, a horse radish peroxidase- (HRP-) conjugated FXIII is cross-linked to the captured FXIIIa. The cross-linked HRP is then detected with a HRP chromogenic substrate at 450 nm. The data was analyzed to evaluate the correlation between FXIII activity and other DIC markers which included platelet count, fibrinogen activities, prothrombin time (PT), activated partial thromboplastin time (APTT), and fibrin degradation product (D-dimer). PT, APTT, fibrinogen, and D-dimer results were generated with an automated clotting analyzer (STA-R).

### 2.5. Genetic Testing

For the genetic testing, a sample of 10 mL 0.05 M EDTA-anticoagulated blood was taken. DNA was extracted according to a standard salting-out procedure. For DNA analysis, commercially available kits were used and the procedure was performed in accordance with the manufacturer's instructions. Mutation status (normal, heterozygous, or homozygous) was determined for all mutations. The analysis of factor V Leiden and prothrombin G20210A mutations was done. For determination of the mutation status of the MTHFR C677T and A1298C mutations, we used PCR amplification and restriction fragment length polymorphism analysis that was performed according to Kim et al., Frosst et al. [9, 10], Frosst et al., and van der Put et al. [10, 11], respectively.

### 2.6. Statistical Analysis

Since the control group has less than 30 patients, all parameters in the study were assumed nonparametric and the statistical analyses were chosen for nonparametric tests. Nominal parameters were described using frequency analysis, whereas scale parameters were described using mean and standard deviations. Comparison between groups was tested by Mann-Whitney* U* at 95% confidence interval (CI; *α* = 0,05 level). All analyses were performed using SPSS 17.0 for Windows (SPSS Inc. Illinois).

## 3. Results

Gender distribution, age, BMI, smoking, diabetes mellitus, rheumatoid factor positivity, hypertension, family history, long standing duration, and birth of more than one child for patient and control groups are gathered as baseline characteristics of the respondents.  Table 1 shows the baseline characteristics of the study and the control group.

The ages of the forty-nine subjects with varicose veins included in the study group ranged between 15 and 85 years (mean 44.04 ± 15,05 years) and the ages of the sixteen subjects in the control group ranged between 22 and 68 years (mean 40,94 ± 13,60 years). There was no statistically significant difference between the ages of the two groups. The women/men ratio was 30/19 in the study group and 15/11 in the control group.

BMI value of the study group was higher than of the control group. The difference was insignificant. The study group included more smokers which is not statistically significant. Diabetes mellitus, hypertension, family history of varicosis, and birth of more than one child of the study group were higher than of the control group. Differences between the two groups were found statistically insignificant (*P* > 0,05) except for standing for long durations (*P* < 0,001) and rheumatoid factor positivity that were found to be statistically significant (*P* < 0,05). CEAP classification of patients was shown in Table 2. It is possible that increasing severity of C class might result in greater differences in hematological or biochemical parameters. Since the classification of the patient group in the study was most notably between class 2 (C2) and class 3 (C3), the differences were not noted in this study with the increasing C class. According to Table 2, CEAP class of the study group was changing between class 2 (C2) and class 5 (C5). Comparison of hematological and biochemical parameters in the study and the control groups was given in Table 3.

No statistically significant difference was observed between both groups with respect to haematological and biochemical parameters, including whole blood count, erythrocyte sedimentation rate, prothrombin time, partial thromboplastin time, C-reactive protein level, glucose level, cholesterol profile, electrolyte levels, liver and kidney function tests, homocysteine, vitamin B12, folic acid, factor V, factor VIII, and protein C and protein S levels.  Table 4 shows the comparison of point mutations in the study group and the control group.

When we look at the comparison of point mutations between the study group and the control group, heterozygote mutations were more dominant in the study group, but the difference was not statistically significant. Homozygote mutations for all parameters were seen to be rare and scattered in both study and control groups. We could say that heterozygote mutations were dominant in the study group compared to the control group, but the difference was not statistically significant.

## 4. Discussion

Incidence of varicose veins in adult population has been shown to vary among populations (between 10% and 60%) and to increase by age in various studies [1–6]. The main factors in the etiology of varicose vein are venous dilation and valvular insufficiency that are started by unknown factors [1–3, 7, 8].

On the basis of experimental and clinical evidence collected for the past decade, it is possible to suggest that the cause of dilatation of the varicose vein is in the vascular wall [12, 13]. The “valvular” theory, suggesting valvular incompetence, has been criticized in a number of biochemical and morphological studies [14]. Despite the evidence of a primary defect in the vein wall, the pathogenesis of the vein dilatation remains obscure.

Javien's study showed that varicose veins were more common in women, but female sex was not found to be a strong risk factor. Among the risk factors most closely associated with chronic venous insufficiency (CVI) were age, family history of varicose veins [15], and constipation. This is in keeping with findings from recent epidemiologic studies [16, 17]. Obesity and lack of physical activity were strongly associated with CVI in women, more so than in men. The number of pregnancies significantly distinguished between women with and without CVI. A modest association was found with female sex, previous injury in legs (DVT), and remaining in the standing position for a long time, although these parameters are usually among those mostly agreed on as being risk factors. The role of the prolonged sitting position was not established in this study [16]. We did not also evaluate the prolonged sitting position in our study.

A number of epidemiological studies have shown that, in addition to environmental factors, genetic mechanisms may play a role in determining susceptibility to vascular disease. In particular, abnormalities in the enzymes that control homocysteine metabolism have been shown to cause vascular disease [9].

Methylenetetrahydrofolate reductase (MTHFR) plays an important role in the folate cycle and contributes to the metabolism of the aminoacid homocysteine. It catalyses the reduction of 5,10-methylenetetrahydrofolate to 5-methyltetrahydrofolate, thus generating the active form of folate required for remethylation of homocysteine to methionine. Some of the mutations in the MTHFR gene cause a decrease in MTHFR activity. If there is a genetic mutation in the* MTHFR* gene, homocysteine levels may not be regulated properly. Genetic mutations in* MTHFR* are the most commonly known inherited risk factor for elevated homocysteine levels. The most common* MTHFR* mutation is called the MTHFR C677T mutation, or the “thermolabile”* MTHFR* mutation. Another common mutation is called* MTHFR* A1298C. Even when 2* MTHFR* mutations are present (e.g., 2 C677T mutations, or one C677T mutation and one A1298C mutation), not all people will develop high homocysteine levels. Although these mutations do impair the regulation of homocysteine, adequate folate levels essentially “cancel out” this defect. The MTHFR polymorphism, which is associated with a predisposition for elevated plasma concentrations of homocysteine, has been reported to represent a genetic risk factor for occlusive vascular diseases, carotid atherosclerosis, silent brain infarction, and small artery occlusion with ischemic stroke, although these associations remain controversial [9]. In our study we studied whether these mutations are associated with another type of vascular disease in the form of varicose veins because the observation of vascular complications in patients with homocystinuria led to the hypothesis that mild to moderate elevation of plasma homocysteine may be related to changes in the vascular wall [18]. Whether mild hyperhomocystinuria is causally related to the development of varicose veins was not known. Since genetic mechanisms may play a role in determining susceptibility to vascular disease, we studied several mutations in patients with varicose vein to look for any association between varicose veins and homocysteine level, protein C, protein S, FV, FVIII, D-dimer, vitamin B12 level, folic acid level, MTHFR, FVR2, B fibrinojen, and FV Leiden and prothrombin mutations.

In our study, we did not observe high levels of homocysteine in our varicose disease patients compared to control group. The results of our study showed that there was no statistically significant difference between patients and control groups based on their baseline characteristics except for standing durations of the patients and rheumatoid factor positivity. Age, BMI, gender distribution, HT, family history, and birth of more than one child were not different between groups. Although some researches reported that family history of the patients has an effect on varicose veins [15], we found it noneffective in the study. The reason may be the lack of sampling or other exogenous variables. Mutation rates were different between groups. In addition, it was seen that heterozygote mutation was dominant in both male and female patients in the patient group, but the difference was not significant. A study done on a population of 1684 individuals around Turkey demonstrated that the frequency of the C677T in Turkey was 42.9%; of C677C, 47.4%; and of T677T, 9.6%. The frequency in Turkey of A1298C was 43.7%; of A1298A, 46.3%; and of C1298C, 10.0%. The allelic frequencies of the T allele of MTHFR 677 and the C allele of MTHFR 1298 were 33.34 and 33.16%, respectively. The frequency of C677T/A1298C compound heterozygosity is highest in Turkey (21.6%), as compared to Canada (15%), the United States (17%), and The Netherlands (20%). This might cause the dominance of heterozygosity in our study [19].

Genetic abnormalities specific to factor V, prothrombin, and homocysteine metabolism were shown to increase the risk for myocardial infarction and ischemic stroke, particularly among younger patients and women in a study [20]. Unlike our results Sverdlova AM demonstrated an association between the MTHFR genotype and the risk of developing varicose veins in the lower limbs. They found a significantly higher prevalence of subjects with at least one C677T MTHFR allele among those with varicose veins than among a control group (OR = 1.74; *P* < 0,005) [21]. This was also notedby the study of Wilmanns and colleagues [22]. We did not see any significant relation with varicose vein or MTHFR mutations. This could be because of the undersampling of our study. Studies including higher numbers of cases and controls could show more significant relations between them.

There are some other limitations in our study also. Since the number of cases in the patient and control group is small, we did not take the effect of the type (superficial and deep reflux) and the degree of reflux into consideration in the study. Economical factors and the type of rheumatic diseases were not evaluated in the study also. We only studied rheumatic factor positivity which was found to be statistically significant compared to the control group.

While there was a trend towards an increase in certain biochemical parameters in our study, ultimately there was no statistically significant difference in hematological or biochemical markers between the subject group and the control group. In this small study, there appear to be no significant genetic differences related to folate metabolism or procoagulant predisposition or medical risk factors including BMI value, smoking, diabetes mellitus, hypertension, family history of varicosis, and birth of more than one child. A history of increased standing and rheumatoid factor positivity were found to be associated with varicose veins. Further studies, perhaps with a larger cohort, could show significance of parameters that were found to be insignificant in our study. Further studies should also be conducted to find any association between rheumatic diseases and varicose vein development.

## Figures and Tables

**Table 1:** Demographic and clinical data of patients.

Parameter	Study group	Control group	*P*
Number of patients, *n*	49	16	
Female/male	30/19	15/11	0,111
Gender: female, *n* (%)	30 (61,2)	15 (57,7)	
Age (years; mean ± SD)	44,04 ± 15,05	40,94 ± 13,60	0,514
Body mass index (kg/m^2^, mean ± SD)	29,09 ± 5,03	28,85 ± 4,39	0,858
Smoker, *n* (%)	15 (30,6)	5 (19,2)	0,570
Type 2 diabetes mellitus, *n* (%)	6 (12,2)	6 (23,1)	0,118
**Rheumatoid factor positivity, ** *n * ** (%)**	4 (8,2)	—	**<0,05**
Hypertension, *n* (%)	14 (28,6)	3 (11,5)	0,320
Family history of varicosis (%)	24 (49,0)	4 (15,3)	0,086
**Standing duration >8 hours, ** *n * ** (%)**	42 (85,7)	9 (34,6)	**0,001**
Birth number >1, *n* (%)	13 (26,4)	3 (11,5)	0,214

**Table 2:** CEAP classification of patients.

CEAP class	Study group		Control group	
	Male	Female	Male	Female
C0	—	—	—	—
C1	—	—	—	—
C2	10 (52,6)	12 (40,0)	—	—
C3	6 (31,6)	18 (60,0)	—	—
C4	1 (5,3)	—	—	—
C5	2 (10,2)			
C6	—			

**Table 3:** The comparison of haematological and biochemical parameters in the study and the control group.

Parameters	Study group	Control group	*P*
Hemoglobin (g/dL)	13,8 ± 1,53	11,8 ± 1,64	0,111
Ferritin	29,38 ± 37,06	19,80 ± 5,54	0,495
D-dimer	246,57 ± 148,63	250,20 ± 68,29	0,639
Protein C	106,22 ± 24,58	120,38 ± 37,45	0,673
Protein S	102,56 ± 18,70	99,25 ± 32,04	0,606
Blood glucose	94,97 ± 21,59	112,45 ± 35,80	0,160
Triglyceride	187,08 ± 84,37	150,82 ± 63,24	N/A
Vitamin B12	258,67 ± 42,40	250,40 ± 21,09	0,187
Folic acid	8,07 ± 3,00	11,10 ± 6,64	0,209
Factor V	108,00 ± 27,82	122,25 ± 15,57	0,321
Factor VIII	133,09 ± 26,89	118,63 ± 42,87	0,840
Sedimentation rate (mm/h)	17,00 ± 18,75	17,40 ± 7,06	0,275
MPV	8,37 ± 0,89	8,17 ± 0,75	0,687
PT (s)	10,97 ± 0,76	11,29 ± 1,50	0,531
aPTT (s)	28,30 ± 2,14	26,83 ± 2,86	0,287
LDL cholesterol (mg/dL)	119,84 ± 31,45	112,36 ± 47,73	N/A
Homocysteine	12,43 ± 5,25	11,80 ± 5,37	0,788

**Table 4:** The comparison of point mutations in the study group and control group.

Parameters	Patient group				Control group			
	Male		Female		Male		Female	
	Het	Hom	Het	Hom	Het	Hom	Het	Hom
MTHFR(C677T)	5	*— *	13	1	1	—	6	2
MTHFR(A1298)	1	2	5	—	—	—	—	1
FVR2	—	—	—	1	—	—	—	—
B fibrinogen	2	—	1	—	1	—	—	—
FV Leiden	6	1	2	—	1	—	—	—
Prothrombin	—	—	—	—	—	—	—	2

Het: heterozygote; Hom: homozygote.
